# Polycomb misregulation in enterocytes drives tissue decline in the aging *Drosophila* intestine

**DOI:** 10.1101/gr.281058.125

**Published:** 2026-01

**Authors:** Sarah M. Leichter, Kami Ahmad, Steven Henikoff

**Affiliations:** 1 Basic Sciences Division, Fred Hutchinson Cancer Center, Seattle, Washington 98109, USA;; 2 Howard Hughes Medical Institute, Chevy Chase, Maryland 20815, USA

## Abstract

Aging compromises intestinal integrity, yet the chromatin changes driving this decline remain unclear. Polycomb-mediated repression is essential for silencing developmental genes, but this regulatory mechanism becomes dysregulated with age. Although shifts in Polycomb regulation within intestinal stem cells have been linked to gut aging, the Polycomb landscape of differentiated cell types remains unexplored. Differentiated cells comprise the majority of the gut epithelium and directly impact both tissue and whole organismal aging. Using single-cell chromatin profiling of the *Drosophila* intestine, we identify cell type–specific chromatin landscape changes during aging. We find that old enterocytes aberrantly repress genes essential for transmembrane transport and chitin metabolism, contributing to intestinal barrier decline, an example of antagonistic pleiotropy in a regenerative tissue. Barrier decline leads to derepression of JAK/STAT ligands in all cell types and increased proliferation of aging stem cells, with elevated RNA polymerase II (RNAPII) at S-phase-dependent histone genes. Specific upregulation of histone genes during aging stem cell proliferation resembles RNAPII hypertranscription of histone genes in aggressive human cancers. Our work reveals that misregulation of the Polycomb-mediated H3K27me3 histone modification in differentiated cells during aging not only underlies tissue decline but also mirrors transcriptional changes in cancer, suggesting a common mechanism linking aging and cancer progression.

Aging is associated with the decline of tissue function and is the leading risk factor in a multitude of diseases (Niccoli and Partridge 2012). The chromatin landscape of cells undergoes profound changes during aging, including redistribution of Polycomb-mediated repressive marks such as trimethylation of histone H3 at lysine 27 (H3K27me3) (Emerson and Lee 2023). For example, global gains in H3K27me3 and widespread heterochromatinization have been observed in the livers of aged mice (Yang et al. 2023) and in the muscles of aging *Drosophila* (Ma et al. 2018), leading to transcriptional silencing. Forcing aged mouse liver cells to re-enter the cell cycle partially restores a youthful H3K27me3 landscape, suggesting that DNA replication may rejuvenate chromatin structure (Yang et al. 2023). However, even mitotically active somatic stem cells display age-related increases in Polycomb repression: Aged *Drosophila* intestinal stem cells (ISCs) and mouse hematopoietic stem cells exhibit elevated dimethylation of the H3K27 residue (H3K27me2) and H3K27me3 levels, respectively, and partial differentiation linked to these changes (Sun et al. 2014; Tauc et al. 2021).

These observations suggest that changes in Polycomb-mediated repression contribute to age-associated functional decline. Supporting this, heterozygous loss-of-function mutations in Polycomb group proteins such as the histone methyltransferase Enhancer of zeste (*E(z)*) lead to life span extension (Siebold et al. 2010), implying that Polycomb repression is detrimental in older animals. Yet, Polycomb group proteins are essential for development (Boyer et al. 2006; Blackledge and Klose 2021), and homozygous mutants are lethal (Denell and Frederick 1983), suggesting that although Polycomb activity is beneficial during development, it may become detrimental with age.

Whereas Polycomb-mediated repression appears to underlie age-associated tissue dysfunction, investigations have so far been limited to postmitotic or stem cell systems, overlooking regenerative tissues made up of both stem and differentiated cells. The adult *Drosophila* intestine offers a model for regenerative tissues because the epithelium undergoes continuous cell turnover, with a median cellular life span for differentiated cells of 4 days (Liang et al. 2017). This rapid turnover ensures that both young and aged intestines maintain similar cellular age distributions. Despite this, the *Drosophila* intestine displays a distinctive age-related decline. Key features include barrier dysfunction, which promotes microbial dysbiosis and systemic inflammation (Rera et al. 2011, 2012; Martins et al. 2018; Salazar et al. 2023), as well as an increase in stem cells (Biteau et al. 2008; Rodriguez-Fernandez et al. 2020; Tauc et al. 2021). Tauc et al. (2021) demonstrated that aged ISCs misexpress lineage-specific markers via altered accessibility at Polycomb-regulated loci. However, differentiated cells comprise a majority of the epithelium (Micchelli and Perrimon 2006; Ohlstein and Spradling 2006), and defects in these cells alone have been shown to cause systemic aging (El Maï et al. 2023). Given the rapid turnover of differentiated cells, age-dependent changes in Polycomb-mediated silencing in differentiated cells could be a major driver of tissue dysfunction.

In this study, we aim to test this hypothesis through single-cell profiling of the H3K27me3 mark in the *Drosophila* intestine to distinguish cell types and capture cell type–specific dynamics of Polycomb-associated modifications during aging.

## Results

### Single-cell chromatin profiling of the fly gut

**Figure 1.**
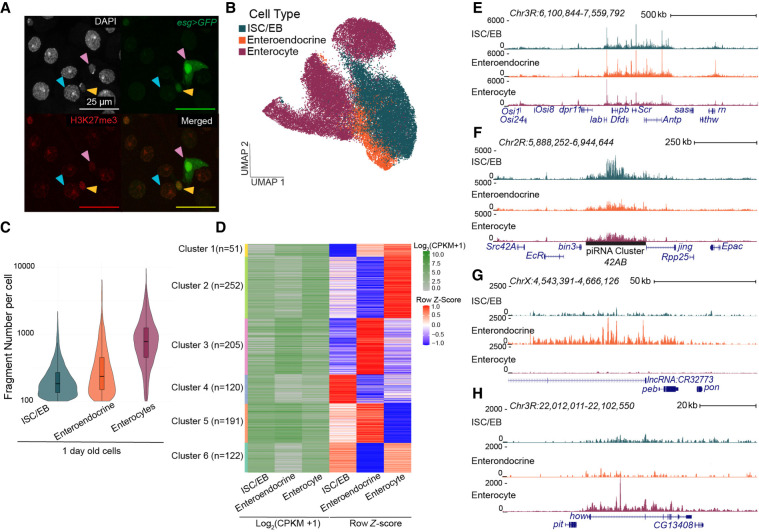
Repressed landscapes for cell types in the *Drosophila* gut. (*A*) Immunofluorescence image of the gut in a young *esgGal4/CyO; UAS-GFP/TM6B* female. GFP (green) marks stem cells and enteroblasts; red anti-H3K27me3, DAPI in gray. Pink arrowheads point to ISC/EBs; blue arrowheads point to enterocytes; and yellow arrowheads point to enteroendocrine cells. Enterocytes are the large DAPI-stained nuclei (blue arrow). Enteroendocrine cells are identified by their small nucleus size in relation to the larger enterocytes and low GFP signal (yellow arrows). (*B*) UMAP of H3K27me3 signal in gut cells from 1-, 15-, and 40-day-old females. Clusters are assigned cell types by a low chromatin silencing score (CSS) at marker genes. (*C*) Violin plots of the distribution of fragments per cell for 1-day-old cell types. (*D*) Log_2_-transformed counts per kilobase per million (CPKM)–normalized and *Z*-score-normalized heatmaps from differential analysis of H3K27me3 over genes between 1-day-old cell types. (*E*–*H*) UCSC Genome Browser tracks of repressed domains in the three cell types in young guts. (*E*) The *ANTP-C* domain is shared between all three cell types. (*F*–*H*) A domain encompassing the piRNA cluster *42AB* is present only in ISC/EBs (*F*), a domain encompassing the *lncRNA:CR32773* gene is present only in enteroendocrine cells (*G*), and a domain encompassing the *how* gene is present only in enterocytes (*H*).

The epithelium of the *Drosophila* adult gut is composed of four cell types: ISCs, enteroblasts (EBs), enterocytes, and enteroendocrine cells (Micchelli and Perrimon 2006; Ohlstein and Spradling 2006). ISCs divide symmetrically to populate the niche or asymmetrically to birth an EB, an undifferentiated progenitor cell that differentiates into an enterocyte or enteroendocrine cell (Micchelli and Perrimon 2006; Ohlstein and Spradling 2006). Enterocytes are the most abundant cell type in the *Drosophila* gut and primarily function in absorption and transportation of nutrients and secretion of digestive enzymes, whereas enteroendocrine cells secrete gut hormones (Hung et al. 2020). Immunostaining of guts from 1-day-old *esg-GAL4*, *UAS-GFP* female flies, which marks ISCs/EBs with a high level of GFP expression, displays distinct cell sizes and levels of GFP expression (Fig. 1A). ISC/EB cell nuclei are small with strong GFP expression, whereas enteroendocrine nuclei are also small with a low level of GFP expression. Finally, enterocytes have large polyploid nuclei with no GFP expression. Additionally, each cell type displays distinct patterns of H3K27me3 staining, implying that gut cell types may be distinguished by chromatin landscapes of this histone modification. We used single-cell combinatorial indexing CUT&Tag (sciCUT&Tag) to barcode cells in a profiling experiment (Janssens et al. 2024). Anticipating that we would compare single cells from different ages, we dissociated guts from female flies of different ages in replicate in one experiment. We chose three ages to span adulthood in *Drosophila*: 1 day after eclosion (young), 15 days after eclosion (middle-aged), and 40 days after eclosion (old-aged). Forty guts from virgin females of each age were barcoded by tagmentation, and then, cells were arrayed on two ICELL8 chips for barcoded PCR (Supplemental Table S1). Peaks were called on the aggregate data for each age using SEACR (Meers et al. 2019) and then merged for dimensionality reduction and uniform manifold approximation and projection (UMAP) embedding in ArchR (Supplemental Table S2; Granja et al. 2021). We applied multiple filtering steps to retain cells that had fragment counts of at least 100 (Supplemental Table S3), a high fraction of reads in peaks (FRiP) (Supplemental Table S4), and a low fraction of blacklisted reads (Supplemental Table S5; Amemiya et al. 2019), which after removing doublets yielded 40,982 cells for analysis.

To assign cell types using a repressive modification, we calculated chromatin silencing scores (CSSs) (Wu et al. 2021), in which a low CSS indicates that a gene is not repressed and could be expressed, and high scores indicate a gene is repressed. CSSs anticorrelate with cell type–specific gene expression profiles (Wu et al. 2021). Using ArchR for clustering and then scoring known marker genes for each cell type (Hung et al. 2020), we identified individual cells as ISC/EBs with low CSS at the escargot (*esg*) gene, enteroendocrine cells at the Piezo gene, and enterocytes at the nubbin (*nub*) gene (Supplemental Fig. S1A). We were unable to separate ISCs from EBs so we group them together as an ISC/EB cluster for subsequent analyses. These three cell types form five distinct clusters on the gut UMAP: one of ISC/EBs, one of enteroendocrine cells, and three clusters of enterocytes (Fig. 1B).

First, focusing on young guts, our analysis includes 11,586 young cells with 68% (7913) of the cells called as enterocytes (Supplemental Table S6). Enterocytes have the highest fragments per cell (Fig. 1C), which might be caused by more widespread repression in this cell type and/or caused by polyploidy of these cells. To identify the genes repressed between cell types, we performed differential analysis for genes that fall within H3K27me3-marked domains as determined using our SEACR peaks (Supplemental Tables S7–S10). H3K27me3-marked domains stretch multiple kilobases in length such as the canonical Antennapedia-Complex (*ANTP-C*), bithorax-complex (*BX-C*), and the Posterior sex combs–Suppressor of zeste 2 (*Psc-Su(z)2*) domains (Schwartz et al. 2006), which are shared across the three cell types (Fig. 1E; Supplemental Fig. S1B,C). There are regions that fall outside of the canonical domains, such as at the rotund (*rn*) gene, that appear to be H3K27me3-marked in enteroendocrines and ISC/EBs but not in enterocytes (Fig. 1E), indicating differences in the H3K27me3 landscape between the cell types. Of the 8697 genes in domains, 10.7% (933) show differential signal between the young cell types from differential analysis (Fig. 1D; Supplemental Table S11). Genes in cell type–specific domains include the *42AB* piRNA cluster (Fig. 1F), which is positively regulated by the H3K27me3 modification in *Drosophila* ovarian stem cells (Akkouche et al. 2025) but is expressed at low levels in the gut (Siudeja et al. 2021). In differentiated cells, the noncoding RNA lncRNA:CR32773 forms an H3K27me3 domain in enteroendocrine cells (Fig. 1G), and held out wings (*how*) is H3K27me3-marked in enterocytes (Fig. 1H). Some genes are marked with the H3K27me3 modification in two cell types but not the third: Enterocytes and enteroendocrine cells share a greater number of common H3K27me3-marked genes with ISC/EBs (122 genes for cluster 6 and 191 genes for cluster 5, respectively) than they do with each other (51 genes for cluster 1) (Fig. 1D). We interpret this pattern as evidence of lineage specification, in which differentiated cells retain H3K27me3 marks from their cell of origin (ISC/EBs) and therefore resemble progenitors more closely than they resemble each other. We conclude that sciCUT&Tag effectively separates the cell types of the gut and identifies repressed domains characteristic of each cell type.

### Gains and losses of repression in aging cells

**Figure 2.**
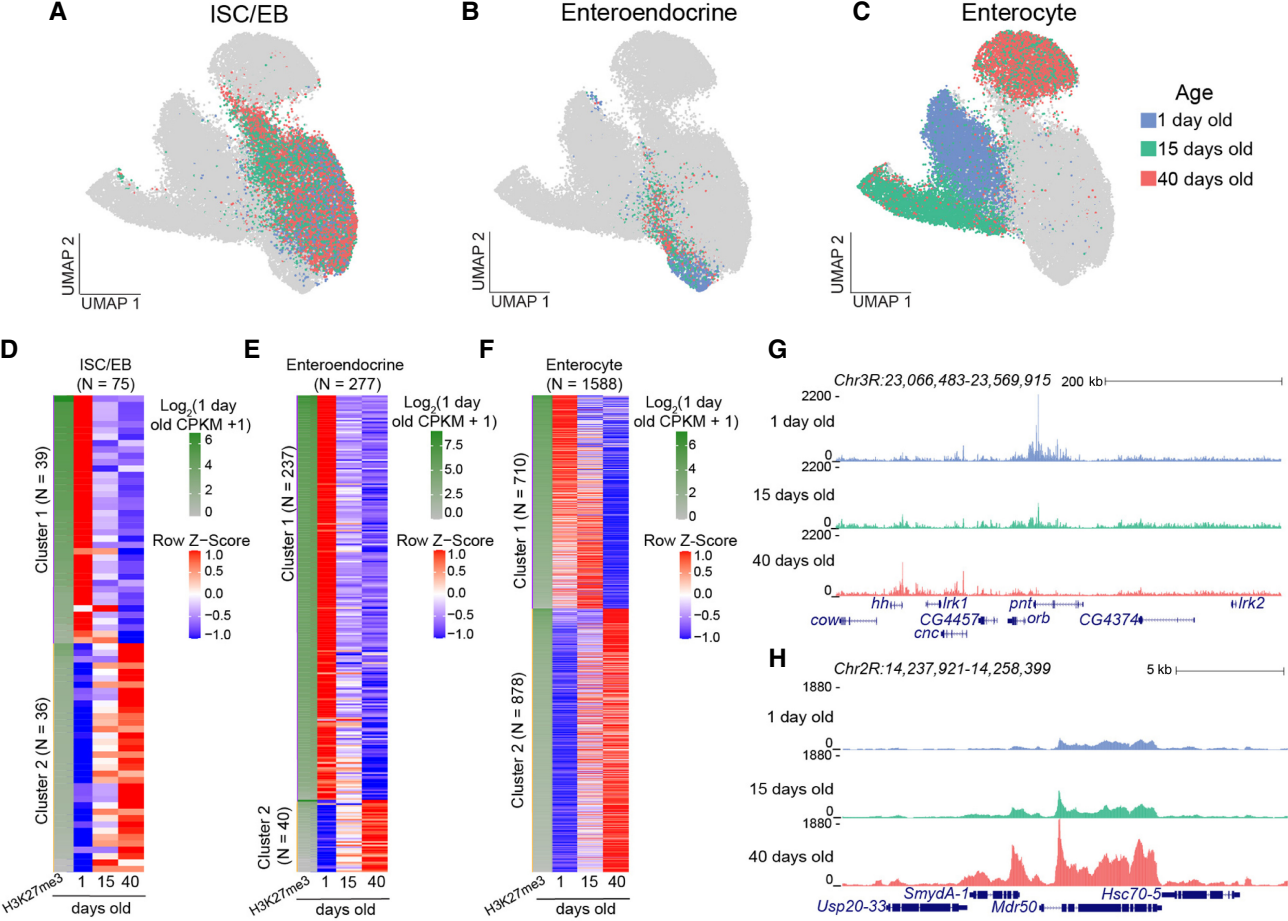
Changes in repressed landscapes in gut cells with age. (*A*–*C*) UMAPs of cell types from guts of different ages. Enteroendocrine cells and ISC/EBs of different ages are mixed within cell type clusters, but enterocytes form three separate clusters. (*D*–*F*) *z*-score-normalized heatmaps from differential analysis of H3K27me3 signal over genes between young and old cells. The first column of each heatmap is the log_2_-transformed CPKM for 1-day-old cells for each cell type. (*D*) ISC/EBs have an almost equal number of genes that lose (39 genes) and gain (36 genes) H3K27me3 signal with age. (*E*) Enteroendocrine cells show a greater number of losses of H3K27me3 signal (237 genes) compared with a gain in H3K27me3 signal (40 genes) with age. (*F*) Enterocytes have the greatest number of genes that show a change in H3K27me3 signal with age (1588 genes) with an age-related gain in H3K27me3 signal in 878 genes. It should be noted that genes that gain H3K27me3 signal are weak domains in young cells, and genes that show a loss of H3K27me3 signal are strong domains in young cells. (*G*,*H*) Genome browser tracks of genes that are differentially methylated in enterocytes, showing a loss (*G*) and gain (*H*) in H3K27me3 signal with age.

Next, coloring the gut H3K27me3 single-cell UMAP (Fig. 1B) based on the three ages of each cell type reveals that ISC/EBs of different ages are intermixed, as are enteroendocrine cells (Fig. 2A,B). This indicates relatively small changes to the chromatin landscapes of these two cell types as animals age. Enterocytes from each age form distinct clusters (Fig. 2C), implying that the chromatin landscape of this cell type changes drastically with age. We also compared fragment counts per cell across ages and lineages, which showed a modest but significant increase with age (Supplemental Fig. S2).

To map age-dependent changes in H3K27me3 landscapes for each cell type, we performed differential analysis between young and old clusters (Supplemental Tables S12–S14). We identified 75 genes in which H3K27me3 changes in ISC/EBs (Fig. 2D), 277 genes in enteroendocrine cells (Fig. 2E), and 1588 genes in enterocytes (Fig. 2F). Among the genes that lose H3K27me3 signal in both ISC/EBs and enteroendocrine cells, GO analysis revealed enrichment for transcriptional regulators and RNA polymerase II (RNAPII)–associated functions (Supplemental Fig. S3A,B; Supplemental Tables S15, S16). This included derepression of multiple transcription factor genes such as worniu (*wor*) in ISC/EBs and Ets at 21C (*Ets21C*) in enteroendocrine cells, which are involved in stem cell proliferation and fate decisions (Supplemental Fig. S3C,D).

In enterocytes, we observed both gains and losses of H3K27me3 with age, in which 710 genes decreased in H3K27me3 signal and 878 increased in H3K27me3 signal. The genes that decreased in H3K27me3 signal with age are strong domains in young cells. For example, a domain encompassing the pointed (*pnt*) and oo18 RNA-binding protein (*orb*) genes loses the H3K27me3 mark (Fig. 2G). In contrast, genes that gained H3K27me3 signal with age are weak domains in young cells. An example of this is the Multidrug resistance 50 (*Mdr50*) gene (Fig. 2H), which encodes an efflux transmembrane transporter expressed in the midgut involved with xenobiotic transport activity and response to insecticide (Denecke et al. 2017). These findings suggest that aging not only weakens pre-existing Polycomb domains but also establishes new repressive marks in differentiated intestinal cells, reshaping the chromatin landscape in a cell type–specific manner.

### Changes in repression at regulatory elements with age

A major advantage of sciCUT&Tag is the ability to directly profile changes at regulatory elements, which single-cell RNA sequencing (scRNA-seq) does not capture. To identify regulatory elements that exhibit changes in H3K27me3 level with age within each cell type, we identified self-transcribing active regulatory region sequencing (STARR-seq) enhancers (Zabidi et al. 2015) that fell within our list of H3K27me3 peaks, acknowledging that this list may miss gut-specific sites. From differential analysis, we identified 18 enhancers that changed with age in ISC/EBs and 406 in enterocytes but none in enteroendocrine cells (Supplemental Fig. S4A,B; Supplemental Tables S17, S18).

Next, we searched for transcription factor motifs within enhancers that significantly change within each cell type using FIMO (Grant et al. 2011) against the JASPAR insect transcription factor database (Rauluseviciute et al. 2024). We find in aged ISC/EBs that enhancers that lose H3K27me3 are enriched for motifs linked to chromatin organization and developmental regulation (e.g., chromatin-linked adaptor for MSL proteins [CLAMP], snail [sna], buttonhead [btd], and Kr transcription factor [Kr]) (Supplemental Fig. S4C). In contrast, those that gain H3K27me3 signal show motifs for developmental regulators such as twist (twi), hb transcription factor (hb), and tinman (tin) (Supplemental Fig. S4D). In enterocytes, motifs for stress-responsive TFs and motifs for Trithorax-like (Trl) and grainy head (grh), which are known to influence enhancer accessibility, dominate in enhancers losing H3K27me3 signal (Supplemental Fig. S4E). At the same time, architectural proteins such as CTCF and Motif 1 Binding Protein (M1BP) are enriched in enhancers that gain H3K27me3 signal with age (Supplemental Fig. S4F). Notably, CLAMP motifs appear consistently across cell types, enriched in enhancers that both lose and gain H3K27me3 signal with age. CLAMP has been shown to displace Trl/GAF during stress and act as a transcriptional repressor (Aguilera et al. 2025). Together, these findings suggest that CLAMP functions in a context-dependent manner during aging, contributing to both repression and derepression of enhancer activity under stress-associated conditions. These results point to chromatin-level changes that may prime these cells for altered transcriptional programs with age.

### Polycomb repression of barrier membrane genes in aging enterocytes

**Figure 3.**
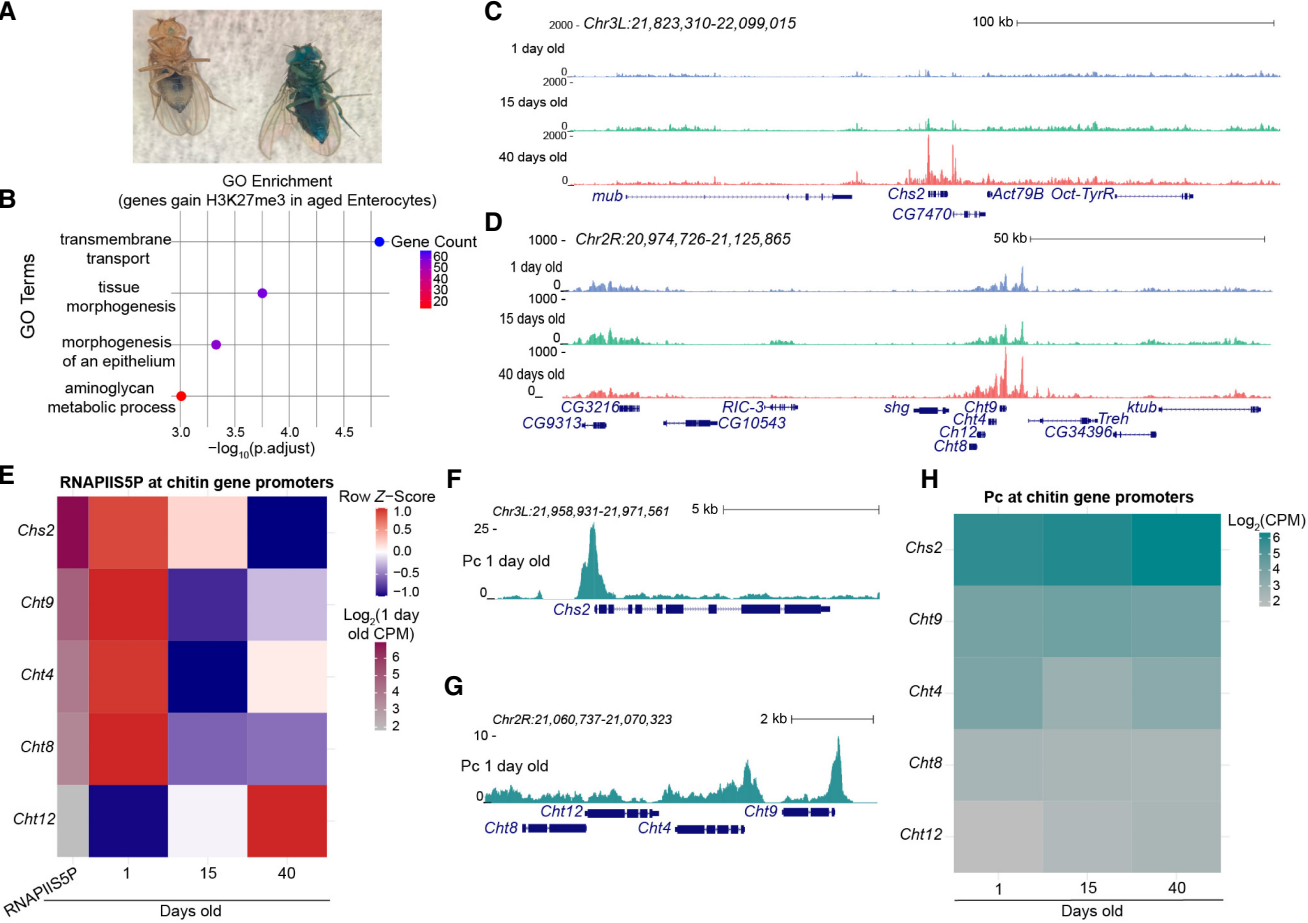
Repression of barrier genes in aged enterocytes. (*A*) Results of a Smurf assay in which flies are fed food containing a nonabsorbable blue dye. Fly on the *left* is 1 day old and has the blue dye is restricted to the digestive tract. Fly on the *right* is 40 days old, and the blue dye can be seen throughout the entire body. (*B*) Top four GO terms on genes that gain H3K27me3 signal in aged enterocytes from differential analysis. (*C*,*D*) UCSC Genome Browser tracks of chitin synthesis (*C*) and chitinase (*D*) genes that form new H3K27me3 domains in aged enterocytes. (*E*) Row *z*-score-normalized heatmap of RNAPIIS5P counts over chitin synthesis gene promoters. The first column of each heatmap is the log_2_-transformed counts for 1-day-old tissue for each promoter. Row *z*-scoring is used to present directionality of the change in signal with age. (*F*,*G*) UCSC Genome Browser track of Polycomb in 1-day-old guts at the promoter of the *Chs2* gene (*F*) and at *Cht9* (*G*). (*H*) Heatmap of log_2_-transformed counts for Polycomb binding at each chitin synthesis gene promoter.

A major defect of the aging *Drosophila* gut is breakdown of the protective barrier membrane (Salazar et al. 2023). This is observed through the “Smurf assay” in which flies are fed food containing a nonabsorbable blue dye that stains only the digestive tract of young flies in which the intestinal barrier is intact, but in old flies, the blue dye spreads through the whole body owing to increased permeability of the gut (Fig. 3A; Rera et al. 2011, 2012; Martins et al. 2018). Given that elevated H3K27me3 levels have been associated with reduced life span (Siebold et al. 2010; Ma et al. 2018), we hypothesized that age-related changes in the H3K27me3 landscape of enterocytes may contribute to barrier dysfunction.

To investigate this, we performed GO analysis of genes that gain or lose H3K27me3 signal in aged enterocytes (Supplemental Tables S19, S20). Among the genes that lose H3K27me3 signal with age, top GO terms include “neurogenesis,” “regulation of transcription by RNA polymerase II,” “generation of neurons,” and “transcription by RNA polymerase II” (Supplemental Fig. S5A), which resulted in a list of transcription factor genes involved in development and differentiation that become derepressed with age (Supplemental Fig. S5B). Notably, this group includes the Enhancer of split (*E(spl)*) complex genes, located on Chromosome 3R, which are normally Polycomb-repressed in young enterocytes (Supplemental Fig. S5C; Josserand et al. 2023), as well as the HOX transcription factor paired (*prd*) (Supplemental Fig. S5D). These findings mirror the chromatin derepression of transcription factor genes in aged ISC/EBs and enteroendocrine cells, consistent with a widespread relaxation of lineage-specific repression programs during aging.

In contrast, GO analysis of genes that gain H3K27me3 signal in aged enterocytes reveals enrichment for the terms “transmembrane transport,” “tissue morphogenesis,” “morphogenesis of an epithelium,” and “aminoglycan metabolic process” (Fig. 3B). Of particular interest is this last term, as the barrier that forms over the intestinal epithelium to protect it from contents from the lumen, the peritrophic matrix, is composed of the aminoglycan chitin (Lehane 1997). The peritrophic matrix of insects, which is analogous to the mammalian mucus-based membrane of the digestive track (Konno and Mitsuhashi 2019), is produced in the foregut, and midgut enterocytes produce chitin to maintain the peritrophic matrix (Dutta et al. 2015; Zhu et al. 2024). Disruption of chitin secretion from enterocytes to the peritrophic matrix leads to barrier dysfunction and susceptibility to stress (Katheder et al. 2023). Two gut-specific genes that acquire strong H3K27me3-marked domains in aged enterocytes are Chitin synthase 2 (*Chs2*) and Chitinase 9 (*Cht9*) (Fig. 3C,D). Both of these genes are involved in maintenance of the peritrophic matrix and in the midgut are expressed in enterocytes (Supplemental Table S21; Buchon et al. 2013; Hung et al. 2020). Degradation of chitin and silencing of *Chs2* are key targets for the development of insecticides against pests such as mosquitos (Zhang et al. 2023). *Cht9* is specifically expressed in the gut (Leader et al. 2018; Krause et al. 2022) but is clustered together with three other chitinases (Chitanse *8* [*Cht8*], Chitinase 12 [*Cht12*], and Chitinase 4 [*Cht4*]) that all become marked with H3K27me3 with age (Fig. 3D). The H3K27me3 domains only form over *Chs2* and the *Cht4-8-9-12* chitinase gene clusters in aged enterocytes, implying that they become repressed in older animals (Supplemental Fig. S6A,B).

To determine if the increase in H3K27me3 signal across the domain leads to transcriptional silencing of *Chs2* and *Cht9*, we profiled the initiating serine-5-phosphorylated isoform of RNAPII (RNAPIIS5P) in whole guts using CUT&Tag under CUTAC low-salt tagmentation conditions (Supplemental Tables S1, S22; Henikoff et al. 2020; Janssens et al. 2022). RNAPIIS5P provides a direct view of ongoing transcriptional engagement and promoter-proximal regulation that RNA-seq does not capture.

Previously published scRNA-seq data from the aging *Drosophila* intestine (Tauc et al. 2021) were generated from FACS-isolated cells, resulting in data sets heavily enriched for ISCs and EBs, with few differentiated cells represented (Supplemental Table S23). In contrast, our single-cell H3K27me3 and bulk RNAPII-S5P data sets were generated from whole guts without cell sorting. Because of these fundamental differences in sample composition and experimental design, the published scRNA-seq data are not directly compatible for integration with our H3K27me3 sciCUT&Tag data set.

We performed four replicates of RNAPIIS5P CUTAC per age and identified a high Pearson's correlation between replicates within an age group, allowing us to merge the four replicates for analysis (Supplemental Fig. S7). We found that RNAPIIS5P is present at the promoters of the *Chs2*, *Cht4*, and *Cht9* genes in young tissues and is lacking at *Cht12*, which is not expressed in this tissue (Leader et al. 2018; Krause et al. 2022). For the three genes that are expressed, signal decreases with age (Fig. 3E; Supplemental Fig. S8A,B), confirming that these genes become repressed. From differential analysis between young and old guts, *Chs2* and *Cht9* are significantly repressed with age (Supplemental Table S24).

We next asked why H3K27me3 would accumulate over chitin genes with age. The H3K27me3 mark is bound by the Polycomb chromodomain protein (Pc) (Cao et al. 2002), but Pc is also localized at the promoters of many active genes (Orsi et al. 2014). Given the duality of Pc binding, we profiled the Pc protein in all three ages of whole guts by CUT&Tag (Supplemental Tables S1, S25). We found that Pc binds promoters both within and outside of H3K27me3-marked domains identified across all three cell types and ages. In the intestine, a larger fraction of promoters fall within H3K27me3-marked domains because cells of this tissue exhibit more broadly repressed chromatin than previously profiled *Drosophila* tissues (Supplemental Fig. S9A,B). Genome-wide analyses have shown that the majority of Pc binding sites in other *Drosophila* tissues are located outside of H3K27me3-marked domains (Orsi et al. 2014; Loubiere et al. 2016; Brown et al. 2018). Promoter-proximal Pc sites outside of H3K27me3 domains differ from Pc occupancy within domains, in which Pc is predominantly anchored at Polycomb response elements (PREs).

We observe minimal changes in Pc signal across ages (Supplemental Fig. S9A,B). For example, the H3K27me3 domain around the gene senseless-2 (*sens-2*) is present in all three cell types and contains multiple Pc binding sites that are stable with age (Supplemental Fig. S9C). In contrast, Sphingosine-1-phosphate lyase (*Sply*) lacks the H3K27me3 mark and is therefore not located within a domain but is bound by Pc (Supplemental Fig. S9D), consistent with prior reports that Pc can localize independently of H3K27me3 (Schaaf et al. 2013; Orsi et al. 2014; Loubiere et al. 2016).

When we examined Pc level at the promoters of chitin genes, we found that it is present around the active promoters of the *Chs2* and *Cht9* genes in young guts (Fig. 3F,G) and in older tissues (Fig. 3H; Supplemental Fig. S8C,D), with high specificity compared with the promoters of genes lacking Pc signal, such as the promoters of the genes radish (*rad*) and dusky (*dy*) (Supplmental Fig. S8E,F). Although the signal at *Cht9* appears low, it exhibits a level of signal greater than IgG, which is a nonspecific antibody that serves as a control for CUT&Tag (Supplemental Table S26; Kaya-Okur et al. 2019, 2020). In a coverage heatmap ordered by Pc descending signal, we find *Chs2* and *Cht9* to be present in the top 25% of promoters within H3K27me3 domains (Supplemental Fig. S9A). Thus, although the presence of Pc at these promoters is compatible with gene expression in young tissues, in aged guts the binding of the Pc chromodomain to H3K27me3 leads to chromatin compaction and gene repression (Kassis et al. 2017). Note that we find that many Pc binding sites do not trigger the formation of a H3K27me3 domain with age (Supplemental Fig. S9B,D). Therefore, we hypothesize that using Pc for gene regulation early in life comes with the risk of nucleating repression later in life.

### Derepression of enteroendocrine genes in aged guts

**Figure 4.**
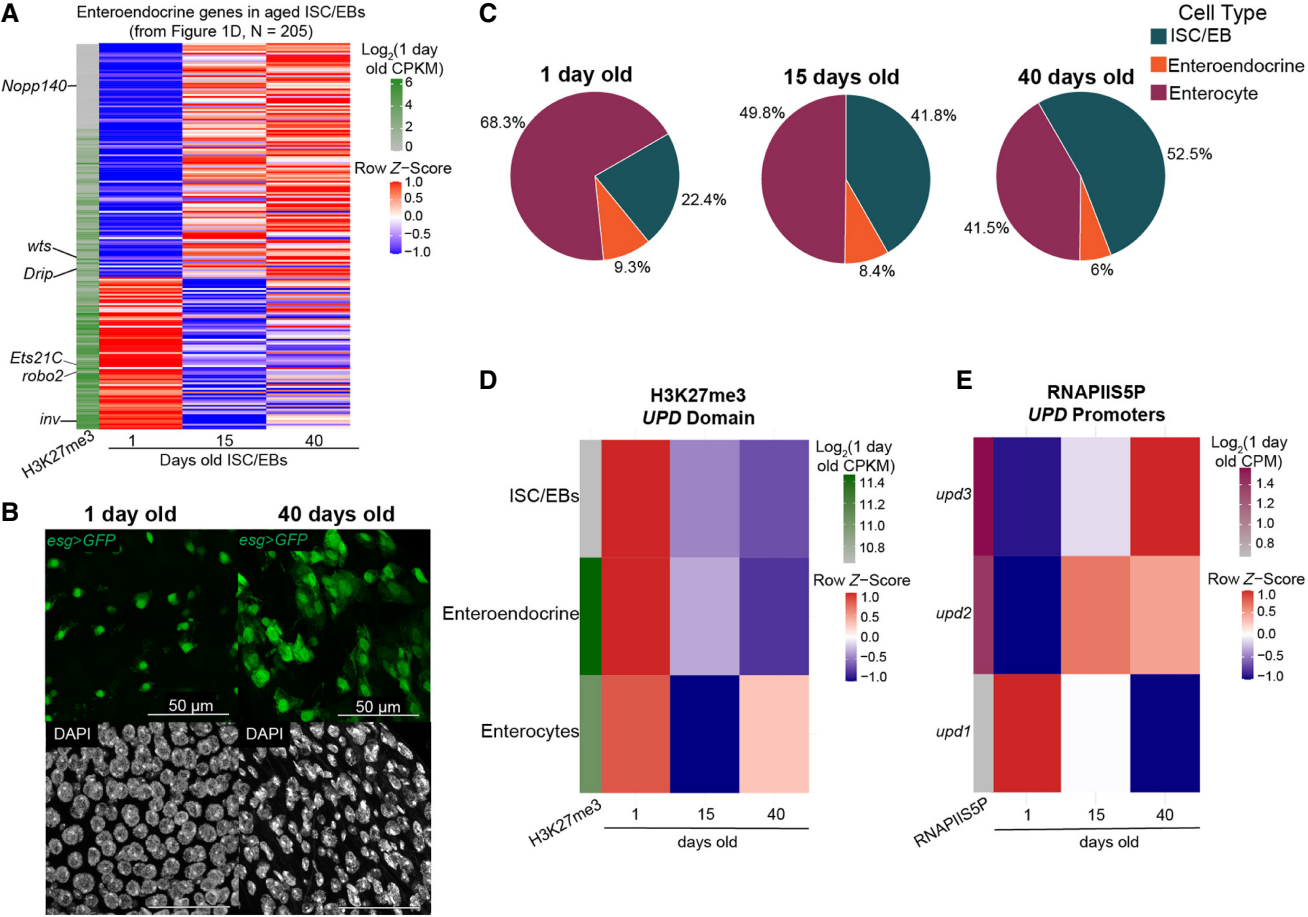
Stem cells display overproliferation and derepression of lineage-specifying genes and JAK/STAT ligands. (*A*) Row *z*-score-normalized heatmap of H3K27me3 counts of ISC/EBs for enteroendocrine genes defined in Figure 1D. The first column is the log_2_-transformed CPKM for 1-day-old tissue for each gene. (*B*) Immunofluorescence image of the gut in a young (1-day-old) and old (40-day-old) *esgGal4/CyO; UAS-GFP/TM6B* female. GFP (green) marks stem cells and EBs; gray, DAPI. (*C*) Pie charts of the proportion of each cell type per age. Significance was assessed by Monte Carlo permutation test (10,000 permutations). Observed enrichment was greater than expected by chance (*P* < 0.0001). (*D*) Row *z*-score-normalized heatmap of H3K27me3 counts over the *unpaired* domain, which encompasses three genes encoding JAK/STAT ligands. The first column is the log_2_-transformed counts for 1-day-old tissue for each promoter. ISC/EBs have the lowest H3K27me3 level of the three cell types in 1-day-old tissue and lose H3K27me3 signal with age, indicating derepression. Row *z*-scoring is used to present directionality of the change in signal with age. (*E*) Row *z*-score-normalized heatmap of RNAPIIS5P counts over promoters of the *upd1*, *upd2*, and *upd3* genes. The first column is the log_2_-transformed counts for 1-day-old tissue for each promoter. *upd2* and *upd3* gain RNAPIIS5P signal in aged tissues, which is consistent with previous reports of these ligands. Row *z*-scoring is used to present directionality of the change in signal with age.

Having established that age-dependent H3K27me3 gain in enterocytes drives barrier failure, we next asked how Polycomb changes in the aged ISC/EB lineage. Previous studies have reported an age-related increase in enteroendocrine precursor cells, identified by coexpression of ISC and enteroendocrine markers, which is linked to changes in chromatin accessibility of Polycomb-regulated loci (Tauc et al. 2021). Using the set of genes marked by H3K27me3 in young enteroendocrine cells from differential analysis (Fig. 1D), we find that aged ISC/EBs gain H3K27me3 signal over many enteroendocrine-marked genes (124 of 205), including Nopp140, warts (*wrts*), and Drip (Fig. 4A), indicating that aged ISC/EBs begin to acquire an H3K27me3 landscape that partially resembles that of enteroendocrine cells, although these cells still cluster with ISC/EBs in our UMAP (Fig. 2A). We also observed the loss of signal at a subset of H3K27me3-marked enteroendocrine genes in aged ISC/EBs, including transcription factors such as *Ets21C* and invected (*inv*), as well as the slit receptor *roundabout 2* (*robo2*) (Fig. 4A). *Robo2* restricts ISC commitment to the enteroendocrine lineage through repression of prospero (Biteau and Jasper 2014). The loss of H3K27me3 at *robo2* in aged progenitor cells may reflect a compensatory mechanism that preserves some stem cell identity by restricting full differentiation into the enteroendocrine lineage. This suggests that aged ISC/EBs adopt a chromatin landscape partially resembling that of enteroendocrine cells, consistent with the model in which aged stem cells exhibit partial differentiation toward the enteroendocrine fate (Tauc et al. 2021). To further support this conclusion, we examined additional genes in aged ISC/EBs that have been identified as markers of enteroendocrine precursors and neural stem cell potential, including *sna*, *wor*, and deadpan (*dpn*) (Tauc et al. 2021). These genes lose H3K27me3 signal in aged ISC/EBs (Supplemental Fig. S10), reinforcing the model that aging pushes progenitor cells toward partial differentiation into the enteroendocrine lineage.

### JAK/STAT ligands become derepressed in aged gut

Overproliferation of stem cells (Biteau et al. 2008) is reflected in the higher abundance of ISC/EBs in older guts (Fig. 4B). Indeed, the proportion of stem cells based on single-cell profiling increases from 22.4% in young tissues to 52.5% in aged tissues (Fig. 4C). Stem cell overproliferation is stimulated by the production of unpaired ligands that activate the JAK/STAT pathway (Jiang et al. 2009). We observe that this domain loses the H3K27me3 mark in all three cell types (Fig. 4D; Supplemental Fig. S11A). This loss is accompanied by an increase in RNAPIIS5P signal at the unpaired 3 (*upd3*) promoter, which is statistically significant by differential analysis (Fig. 4E; Supplemental Fig. S11B; Supplemental Table S24), and the unpaired gene is specifically expressed in the gut (Jiang et al. 2009). The unpaired 2 (*upd2*) promoter also shows increased RNAPII-S5P with age (Fig. 4E; Supplemental Fig. S11C). In contrast, the unpaired 1 (*upd1*) promoter maintains very low RNAPII-S5P at all ages, consistent with persistent H3K27me3 signal over the *upd1* gene in all ages and cell types (Supplemental Fig. S11A,D). Expression of these ligands is known to be regulated by Polycomb silencing, as knockdown of Polycomb silencing components leads to loss of the H3K27me3 mark over a domain encompassing the three unpaired genes, resulting in increased expression and to tumor formation (Parreno et al. 2024). Additionally, we observe increased RNAPIIS5P at the promoters of the JAK/STAT target genes Zinc finger homeodomain 1 (*zfh1*) and 2 (*zfh2*) (Supplemental Fig. S11E,F). These results support the idea that as the gut ages, damaged enterocytes induce derepression of JAK/STAT ligands, leading to overproliferation of stem cells and hyperplasia of the tissue.

### S-phase-dependent histone gene upregulation with cell proliferation

**Figure 5.**
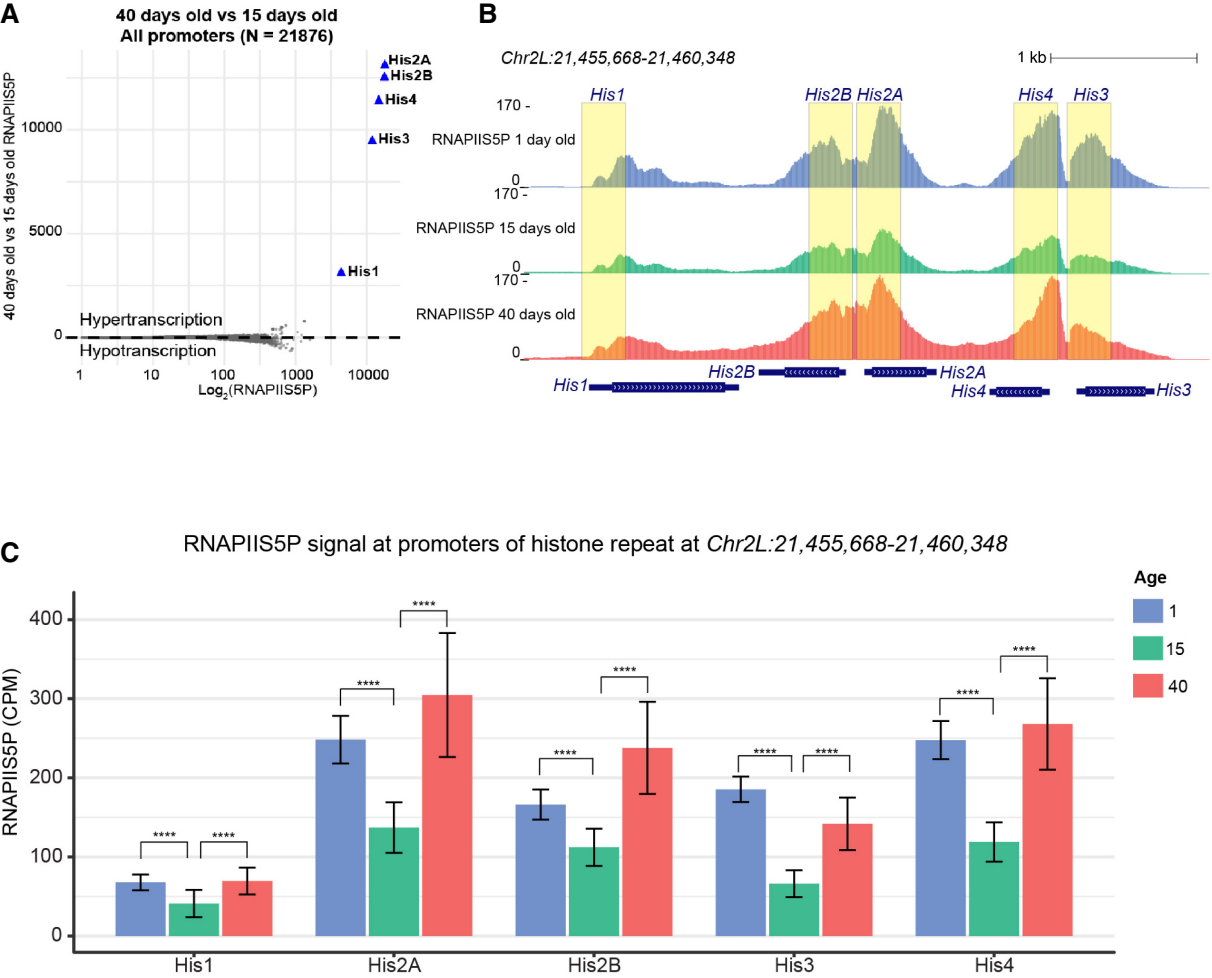
S-phase histone genes are upregulated with global downregulation in aged guts. (*A*) RNAPIIS5P counts for the absolute difference (40-day-old – 15-day-old) counts versus average count (log_10_(40-day-old + 15-day-old)/2) for all promoters in the *Drosophila* genome. (*B*) UCSC Genome Browser track of RNAPIIS5P at a representative histone gene repeat. Yellow boxes highlight the promoter region of each histone gene. (*C*) Bar charts depicting RNAPIIS5P counts per million (CPM) for each promoter of the histone gene repeat shown in *B*. Significance was assessed by a Poisson rate test compared to 15-day-old samples. (*) *P* < 0.05, (**) *P* < 0.01, (***) *P* < 0.001, and (****) *P* < 0.0001.

The increase in ISC/EB cell number prompted us to test for global gene upregulation, or hypertranscription, a general feature of mammalian stem cell proliferation (Kim et al. 2023, 2024). We previously showed that hypertranscription can be detected in cancer by plotting the difference in RNAPIIS5P signal between tumor and normal cells as a function of the average RNAPIIS5P signal (Henikoff et al. 2025). We applied the same analytical approach to *Drosophila* gut samples, for each promoter plotting the RNAPIIS5P difference between each age as a function of the average signal (Altman and Bland 1983), displayed on a log_10_ scale for clarity. Excluding histone genes, neither hyper- nor hypotranscription was observed for the 15- to 1-day comparison (Supplemental Fig. S12A), although hypotranscription was detected between the 40- and 15-day-old and the 40- and 1-day-old guts (Fig. 5A; Supplemental Fig. S12B). The multiple-copy histone genes were exceptional, displaying enormous enrichment of RNAPIIS5P at the multiple-copy S-phase-dependent core histone genes, as well as greater enrichment for Histone H2A (*His2A*), Histone H2B (*His2B*), Histone H3 (*His3*), and Histone H4 (*His4*) than for all 21,876 other annotated promoters (Fig. 5A; Supplemental Fig. S12A,B). In contrast, Histone H1 (*His1*), the linker histone responsible for primary chromatin compaction, showed lower level of enrichment with age (Fig. 5B; Supplemental Fig. S12B). Importantly, S-phase-dependent histone transcripts lack polyadenylation (Marzluff and Koreski 2017) and are poorly captured by standard RNA-seq, making RNAPIIS5P profiling especially suitable for assessing their transcriptional regulation. Direct comparison of 40-day-old versus 15-day-old tissues shows widespread loss of RNAPIIS5P at most genes, accompanied by orders-of-magnitude higher levels at histone loci (Fig. 5B,C). Notably, 15-day-old tissues correspond to the peak of gut maturation, after which age-related decline begins (Capo et al. 2019). This establishes S-phase-dependent histone gene upregulation as a transcriptional signature of advanced aging in this tissue.

RNAPII accumulates at histone genes during times of rapid cell proliferation such as in early *Drosophila* embryos (Huang et al. 2021) and in mouse embryonic stem cells at S phase (Mahat et al. 2024) with corresponding reductions genome-wide. As S-phase-dependent histones are rate-limiting for proliferation, their specific accumulation of RNAPIIS5P is consistent with histone gene transcription driving stem cell overproliferation in the gut. Whereas RNAPIIS5P increases at all four core histone genes in old tissue, the genes for histone His2B and for histone His2A increase the most (Fig. 5C,D), suggesting a higher demand for these histones in the aging gut. In yeast, overexpression of core histone genes extends replicative life span, highlighting their potential role in promoting cellular longevity (Feser et al. 2010). Increased RNAPIIS5P at S-phase-dependent histone genes during aging-associated stem cell proliferation resembles similar upregulation at orthologous histone genes in other rapidly dividing cell types.

## Discussion

**Figure 6.**
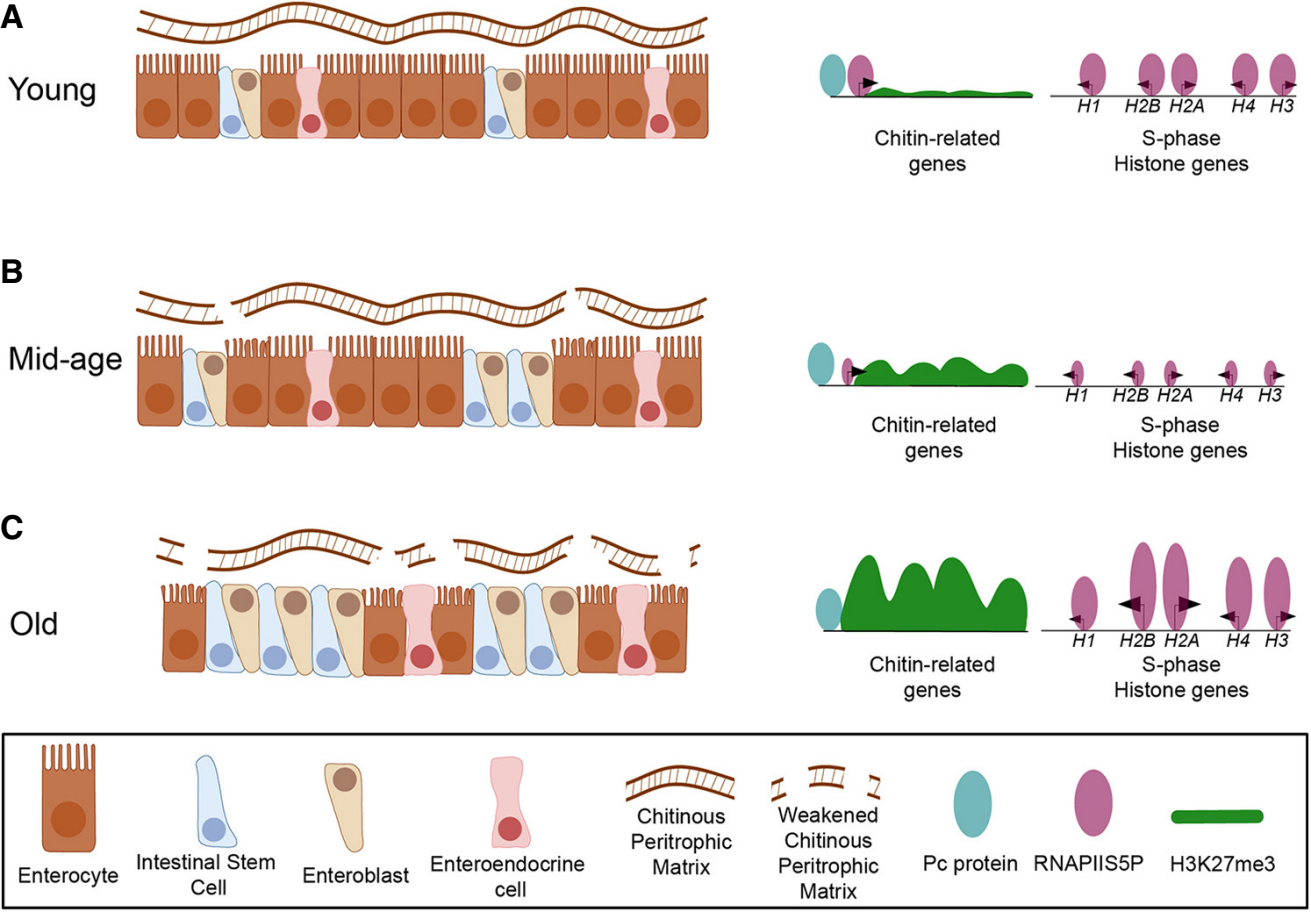
Model for cell type–specific Polycomb remodeling drives gut aging. (*A*) Young gut: The epithelium is composed primarily of enterocytes, with a small population of ISC/EBs. In enterocytes, Polycomb (Pc) binds chitin-related promoters (e.g., *Chs2*, *Cht* cluster), but H3K27me3 levels remain low, allowing robust RNAPII occupancy and continuous peritrophic-matrix production. Stem cells proliferate at homeostatic rates and show low RNAPII occupancy at S-phase histone loci. (*B*) Mid-age gut. Enterocyte numbers decline, and ISC/EBs expand. H3K27me3 signal accumulates at Pc-bound chitin genes in enterocytes, partially repressing chitin synthesis. The peritrophic matrix becomes thinned, causing moderate luminal stress and a slight increase in enterocyte turnover. Stem cells respond with increased proliferation but maintain low RNAPII at S-phase histone genes. (*C*) Old gut. Enterocytes are markedly depleted, and ISC/EBs predominate. In enterocytes, broad Pc-nucleated H3K27me3 domains entirely silence chitin-related loci, weakening the peritrophic matrix and exposing the epithelium to high luminal stress, further reducing enterocyte numbers. Barrier failure triggers stem cell hyperproliferation and massive RNAPII accumulation at S-phase histone loci, a histone hypertranscription signature resembling that of aggressive tumors.

Over a century ago, Elie Metchnikoff hypothesized that systemic aging results from the breakdown of the intestinal barrier (Metchnikoff and Mitchell 1907; Salazar et al. 2023), with toxic effects inducing aging of other tissues (Rera et al. 2012). Our study provides a chromatin-level mechanism for this hypothesis by showing that age-dependent gains of the repressive H3K27me3 mark in enterocytes, and corresponding losses at other loci, alter gene expression programs that underlie gut aging. Enterocytes accrue ectopic H3K27me3 signal over multiple chitin-synthesis genes, compromising the peritrophic matrix that normally shields the epithelium from luminal toxins (Zhang et al. 2023). Consistent with analogous zebrafish studies showing that enterocyte-specific telomerase loss drives systemic aging and that its restoration slows organismal decline (El Maï et al. 2023), our results underscore enterocytes as critical drivers of aging. Although previous *Drosophila* studies attributed gut aging to chromatin changes in ISCs (Tauc et al. 2021), our results position enterocytes as the initiating cell type: By repressing chitin-related genes, aged enterocytes drive barrier failure, stem-cell hyperproliferation, and likely systemic aging effects (Fig. 6). Loss of barrier integrity is not only a hallmark of gut aging but also a strong predictor of imminent death (Rera et al. 2012) as toxins leaking into circulation promote microbial dysbiosis, systemic inflammation, and life span limitation (Clark et al. 2015; Li et al. 2016; Salazar et al. 2023).

Why would chitin synthesis genes acquire Polycomb repression in aged animals? Polycomb repression is mediated by the PRC2 complex, which methylates H3K27 on nucleosomes. The chromodomain of the Polycomb (Pc) protein component of PRC1 specifically binds H3K27me3, but Pc also binds at active promoters (Schaaf et al. 2013; Orsi et al. 2014). We propose that the binding of Polycomb at active promoters carries a risk, at some low frequency, of nucleating repression. Polycomb protein occupies the promoters of both the *Chs2* and *Cht9* genes in guts, and so, as animals age, stochastic nucleation of repression would silence these critical genes, resulting in peritrophic membrane breakdown and leaky guts.

Normal Polycomb regulation in young adult *Drosophila* but aberrant misregulation in aged flies may be a molecular example of antagonistic pleiotropy. In this evolutionary framework, genes beneficial to the organism early in life are selected for, regardless of any deleterious consequences they may have later in life (Williams 1957). This framework explains aging not as the inevitable breakdown of living machinery, or as a necessary programmed stage of life history, but as the unselected side-effect of pleiotropic genes selected for their early benefits. One such example of antagonistic pleiotropy has been documented in the nematode *Caenorhabditis elegans*, in which insulin signaling stimulates reproduction early in life but also shortens life span (Jenkins et al. 2004). In this case, early reproduction is selected for; side-effects on life span are irrelevant. Antagonistic pleiotropy of Polycomb regulation may also explain why genetic reduction of Polycomb components extends life span in *Drosophila* (Siebold et al. 2010), perhaps simply by reducing silencing of chitin synthesis genes in old enterocytes.

We also identified a small number of significant changes to the H3K27me3 landscape in aged ISC/EBs, including derepression of enteroendocrine marker genes, consistent with a prior report of partial differentiation of this cell type with age through Polycomb silencing (Tauc et al. 2021). It is unclear if skewed lineage commitment is a cause or consequence of the accumulation of defective enterocytes in aged tissues. Thus, we attribute aging in the gut to the aberrant Polycomb silencing in enterocytes, which results in stimulation of stem cells to divide, leading to increased RNAPIIS5P occupancy at the histone genes (Fig. 6). Although defective enterocytes may be the proximal cause of gut permeability, the induction of compensatory stem cell proliferation may be responsible for other age-related syndromes.

Notably, cancer risk is strongly associated with age (Havas et al. 2022), and reduced stem cell proliferation reduces this risk (Zhuang et al. 2025). In contrast to cancer, aged guts do not show hypertranscription but rather exhibit a selective increase in RNAPII occupancy at histone genes, likely driven by stem cell hyperproliferation. Other genes appear to lose RNAPII occupancy. The loss at other genes may be owing to changes in cell type proportions with age; however, we cannot confirm this without cell type–specific RNAPIIS5P profiles. Regardless, we observe high levels of RNAPIIS5P at the histone genes in old tissues, suggesting old ISC/EBs overexpress these genes. We previously demonstrated that increased levels of RNAPII at histone genes predict outcome and correlate with whole-arm chromosomal losses in human cancer (Henikoff et al. 2025; Zheng et al. 2025), suggesting a causal role of increased RNAPIIS5P occupancy at the histone genes in cancer development and in aging tissues that exhibit stem cell overproliferation.

## Methods

### Fly husbandry

The *esgGal4/CyO; UAS-GFP/TM6B* strain was a gift from Bruce Edgar. All flies used for chromatin profiling in this study were of the *w*^*1118*^ genotype and were maintained at 25°C. Virgin females were collected and kept in uncrowded conditions of 15 or fewer flies per vial, being flipped every other day to maintain adequate food supply.

### Antibodies

Primary antibodies are as follows: anti-H3K27me3 (Cell Signaling Technologies 9733, lot 19), anti-RNAPIIS5P (Cell Signaling Technologies 13523, lot 3), anti-Pc (a gift from Judith Kassis), anti-IgG (Abcam ab46540), and anti-GFP (Thermo Fisher Scientific 3E6). Secondary antibodies are as follows: guinea pig α-rabbit antibody (Antibodies online ABIN101961, lot 46671), anti-mouse-FITC (Jackson Immuno Research 115-095-166), and anti-rabbit-Texas Red (Jackson Immuno Research 111-585-144).

### Imaging immunostained guts

Guts from 1-day-old or 40-day-old adult females of genotype *esgGal4/CyO; UAS-GFP/TM6B* were dissected, incubated in Accutase (Stem Cell Technologies 07922) for 10 min, and fixed in 4% formaldehyde/PBS with 0.1% Triton X-100 (PBST) for 10 min. They were incubated in 0.3% sodium deoxycholate/PBST 2× for 10 min twice (Lim and Fuller 2012) and then incubated with primary antibodies (1:100 dilution) in goat serum (Fisher Scientific 16-210-072) in PBS buffer overnight at 4°C and then with fluorescently labeled secondary antibodies (1:200 dilution, Jackson ImmunoResearch) for 2 h at room temperature. Guts were stained with 0.5 µg/mL DAPI/PBS and mounted in 80% glycerol on slides and imaged by epifluorescence on Leica Stellaris8 Confocal Microscope at the Fred Hutch Cellular Imaging Shared Resource and processed using Fiji software (Schindelin et al. 2012).

### Smurf assay

The Smurf assay was conducted as previously described (Martins et al. 2018). Smurf food was prepared by adding nonabsorbable blue dye (FD&C blue no. 1, Sigma-Aldrich 861146) to standard fly food at a final concentration of 2.5% (w/v). Virgin female flies were collected and maintained at a density of 20 flies per vial. Flies were monitored daily for signs of “Smurfness” (blue dye outside of the digestive tract indicating intestinal barrier failure) and survival. Flies were flipped daily on to fresh Smurf food.

### SciCUT&Tag library preparation

Forty midguts per age were dissected in 1× PBS, working 10 at a time and moving into a 1.5 mL tube containing 1× PBS on ice during processing. Once 40 midguts of one age were dissected, they were moved to a Petri dish in a drop of 1× PBS on ice and chopped with a razor. Chopped-up tissue was moved to a tube containing 160 µL of 2 mg/mL collagenase (Millipore Sigma C9407) with 50 mM HEPES and 360 µM CaCl_2_ for 1 h at room temperature with gentle vortexing every 15 min. We then added 10 µL of 0.5 M EDTA to inhibit the collagenase and spun down the samples at 600*g* for 3 min and removed the supernatant. The remainder of the procedure was performed following the sciCUT&Tag protocol described by Janssens et al. (2024). Two iCell8 chips (TaKaRa 640019) were run and sequenced on Illumina NextSeq 2000 using a the P1-100 flow cell at the Fred Hutchinson Cancer Center Genomics Shared Resource.

### Bulk chromatin profiling

We dissected guts from female flies that were 1, 15, and 40 days old in 1× PBS, working 10 at a time and moving into a 1.5 mL tube containing 1× PBS on ice during processing. Four replicates per age per epitope of profiling reactions were performed, with five guts per reaction. Each sample was digested in 50 µL of 2 mg/mL collagenase (Millipore Sigma C9407) containing 50 mM HEPES and 360 µM CaCl_2_ 1 h at room temperature with gentle vortexing every 15 min. After dissociation, 5 µL of concanavalin-A-conjugated magnetic beads (ConA beads, Bangs Laboratories BP531) were added and allowed to bind for 10 min at room temperature. Buffer exchange was performed on a magnetic stand (MACSiMAG Separator 130-092-168). Bead-bound cells were incubated with primary antibody in wash buffer (20 mM HEPES at pH 7.5, 150 mM NaCl, 0.5 mM spermidine, 0.05% Triton X-100, and Roche EDTA-free protease inhibitor) overnight at 4°C, incubated with secondary antibody in wash buffer for 1 h at room temperature, and then incubated with protein-A-Tn5 loaded with adapters (Epicypher 15-1117) in 300-wash buffer (20 mM HEPES at pH 7.5, 300 mM NaCl, 0.5 mM spermidine, 0.05% Triton X-100 with Roche cOmplete protease inhibitor) for 1 h. After one wash with 300-wash+ buffer, samples were incubated in CUTAC-DMF tagmentation buffer (10 mM TAPS, 5 mM MgCl_2_, 20% DMF, 0.05% Triton X-100) for 30 min at 37°C. After tagmentation, samples were washed with TAPS wash buffer (10 mM TAPS, 0.2 mM EDTA). Fragment release was performed in 5 µL 1% SDS supplemented with 1:10 Thermolabile Proteinase K (New England Biolabs P8111S) for 1 h at 37°C followed by 1 h at 58°C. SDS was quenched by addition of 15 µL 6% Triton X-100, and PCR was performed by addition of 2 µL each barcoded 10 mM i5 and i7 primer solutions and 25 µL NEBNext 2× PCR Master mix (New England Biolabs ME541L). Libraries were prepared as previously described (Kaya-Okur et al. 2019; Henikoff et al. 2020) with 14 cycles of PCR with 10 sec combined annealing and extension for enrichment of short DNA fragments. Libraries were pooled by volume by epitope and sequenced in PE50 mode on the Illumina NovaSeq X platform at the Fred Hutchinson Cancer Center Genomics Shared Resource.

### Statistics and computational analyses

#### Single-cell sequencing analysis

Demultiplexing was performed using the sciCTextract custom software (Janssens et al. 2024; https://github.com/mfitzgib/sciCTextract). Adapter sequences were cut using cutadapt 2.9 (Martin 2011) with the following parameters: -j 8 -m 20 -a CTGTCTCTTATACACATCT-A CTGTCTCTTATACACATCT-Z. Sequences were aligned using Bowtie 2 version 2.4.2 (Langmead and Salzberg 2012) to dm6 with the parameters –very-sensitive-local –soft-clipped-unmapped-tlen –no-mixed –no-discordant –dovetail –phred33 -I 10 -X 1000. SAM file created by Bowtie 2 was converted to a BAM file and ran “bedtools bamtobed –bedpe” (Quinlan 2014) on the resulting BAM file. For input into the single-cell analysis software ArchR (Granja et al. 2021), we extracted columns 1, 2, 6, and 7 of the BED files, which correspond to the chr, start, and end, and rewrote the headers to include the barcode. Then, the BED files were further processed by reformatting the fourth column to include just the barcode sequence and removing duplicate reads with the same chromosome, start, stop, and barcode (Janssens et al. 2024). We chose to use the “peakmatrix” option in ArchR to generate the LSI, so to call peaks, we merged BAM files from the two iCell8 wafers for each age and used the pseudobulk as input into SEACR using the stringent parameter and a cutoff of FDR < 0.01 (Supplemental Table S2; Meers et al. 2019). Peaks for each age were merged using “bedtools merge.” We then made the following cutoffs of our data in ArchR: keeping cells with at least 100 fragments/cell, keeping cells with only a FRIP of 0.7 and greater, and removing cells with a fraction of reads that fell into the blacklist (Amemiya et al. 2019) that was greater than 0.2 (Supplemental Tables S3–S5). One hundred fragments per cell represents a sequencing depth of 0.000056× coverage per cell for the *Drosophila* genome, which is greater than what has been reported previously for single-cell CUT&Tag (Wu et al. 2021). We then removed doublets using the ArchR addDoubletScores and filterDoublets functions (Granja et al. 2021). For graph-based clustering of sciCUT&Tag data, we used the approach implemented in Seurat (Butler et al. 2018) and then compared common metrics across clusters, including information content (reads/cell) and gene coverage. To annotate cell types using H3K27me3, we calculated CSSs as previously reported (Wu et al. 2021). CSSs are the gene-level, distance-weighted aggregation of single-cell H3K27me3 signal using the ArchR gene activity/score model, which converts fragment counts in tiles around each gene into a depth-normalized score. For H3K27me3, high coverage indicates a “repressed” status, whereas low coverage indicates “unrepressed.”

Once we had annotated the three cell types (ISCs and EBs were too similar separate into distinct clusters), we exported the barcodes from ArchR and using grep commands and pulled out the fragment information for each cell of each cell type for each age. We then created count tables from the cell type–specific fragment files using “bedtools intersect –c” against genes within SEACR peaks (Supplemental Tables S7–S10). Count tables were uploaded to the Degust server (https://degust.erc.monash.edu/) for differential analysis (Supplemental Tables S11–S14). Normalized counts from the count tables were used to generate heatmaps using ggplot2 (Wickham 2016) in R (R Core Team 2025) for visualization. GO analysis was performed using clusterProfiler (Yu et al. 2012) and visualized using ggplot2 (Wickham 2016) in R (Supplemental Tables S15, S16; R Core Team 2025). BED files from each iCell8 chip were merged and created into normalized count bigwigs by the BEDTools (v2.30) (Quinlan 2014) genomecov command with scale (size_of_reference_sequence/total_counts). Normalized count bigwigs are the fraction of counts at each base pair scaled by the size of the reference sequence so that if the counts were uniformly distributed across the genome, there would be one at each position. Tracks were uploaded to the UCSC Genome Browser and then downloaded as PDF files. For analysis of enhancer regions, a list of curated enhancers from *Drosophila* (Zabidi et al. 2015) was downloaded and then filtered for enhancers that fell within our list of H3K27me3 peaks using “bedtools intersect” (Quinlan 2014). We then created count tables for enhancers within peaks from the cell type–specific fragment files using “bedtools intersect –c.” Count tables were uploaded to the Degust server (https://degust.erc.monash.edu/) for differential analysis (Supplemental Tables S17, S18). Differentially enriched enhancers were then used for motif searching using FIMO (Grant et al. 2011) and a list of known transcription factor motifs from the JASPAR insect database (Rauluseviciute et al. 2024). Heatmaps and dot plots were generated in R (R Core Team 2025) using ggplot2 (Wickham 2016).

#### Bulk sequencing analysis

Adapters were clipped using cutadapt 4.1 with the following parameters: -j 8 ‐‐nextseq-trim 20 -m 20 -a AGATCGGAAGAGCACACGTCTGAACTCCAGTCA-A AGATCGGAAGAGCGTCGTGTAGGGAAAGAGTGT-Z. Paired-end reads were mapped to a repeat masked release r6.30 of the *D. melanogaster* genome obtained from UCSC (http://hgdownload.cse.ucsc.edu/goldenPath/dm6/bigZips/dm6.fa.masked.gz) using Bowtie 2 using the following parameters: ‐‐very-sensitive-local ‐‐soft-clipped-unmapped-tlen ‐‐dovetail ‐‐no-mixed ‐‐no-discordant -q ‐‐phred33 -I 10 -X 1000.

Properly paired reads were extracted from the alignments by SAMtools (v1.14) (Danecek et al. 2021) bamtobed command into mapped fragment BED files. Replicate BED files were merged and created into normalized count bigwigs by BEDTools (v2.30) genomecov command with scale (size_of_reference_sequence/total_counts). Normalized count bigwigs are the fraction of counts at each base pair scaled by the size of the reference sequence so that if the counts were uniformly distributed across the genome there would be one at each position. Comparisons between Pc and IgG signal for promoters in Supplemental Table S25 was performed using normalized count bigwigs using UCSC tools BigWigAverageOverBed (Kent et al. 2010).

Count tables were generated using BED files using “bedtools intersect –c” against a promoter list of the dm6 genome version 31 downloaded from FlyBase. Normalized counts from the count tables were used to generate heatmaps using ggplot2 in R (R Core Team 2025) for visualization (Supplemental Tables S22, S23; Wickham 2016). Aggregate plots with heatmaps were generated using deepTools2 (Ramírez et al. 2016).

## Data access

All raw and processed sequencing data generated in this study have been deposited in the NCBI Gene Expression Omnibus (GEO; https://www.ncbi.nlm.nih.gov/geo/) under accession number GSE291173.

## Supplemental Material

Supplement 1

Supplement 2
