# Supplementary material for: Polycomb misregulation in enterocytes drives tissue decline in the aging Drosophila intestine
Source: Genome Res. 2026 Jan;36(1):102–14. doi: 10.1101/gr.281058.125 (PMC12758387; doi:10.1101/gr.281058.125)
Supplement: Supplement 1 [file Supplemental_Figures.docx]

**Supplemental Figures**


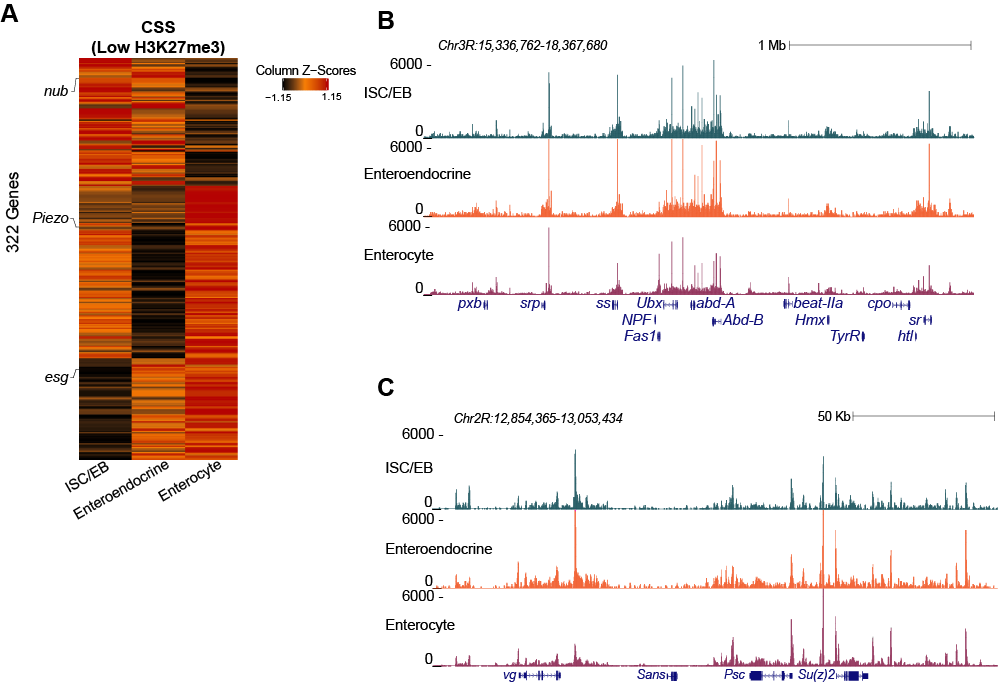


**Supplemental Figure S1**. H3K27me3 sciCUT&Tag**.** (*A*) ArchR heatmap using all cells in the experiment to show chromatin silencing scores (CSS) for genes that have low H3K27me3 signal for each cell type in UMAP in Fig. 1B. (*B-C***)** UCSC Genome Browser tracks of repressed *BX-C* (*B*) and *Psc-Su(z)2* (*C*) domains in the three cell types in young guts.

**Supplemental Figure S2.** Increase in H3K27me3 fragments per cell in aged guts. Violin plots of fragments per cell for three ages of each cell type. P-values represent the results of pair-wise Wilcox tests.

**
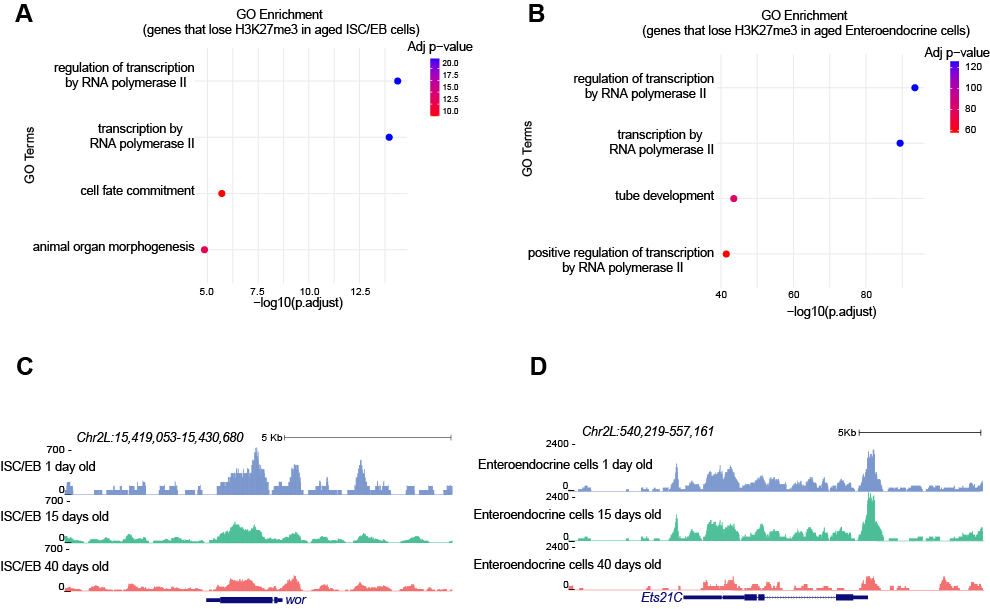
**

**Supplemental Figure S3**. Transcription factor genes lose the H3K27me3 mark in aged ISC/EBs and enteroendocrine cells**.** (*A-B*) Top four GO terms on genes that lose H3K27me3 signal in aged ISC/EBs (*A*) and enteroendocrine cells (*B*) from differential analysis. (*C-D*) UCSC Genome Browser tracks of example genes from GO analysis listed in panels A and B that lose H3K27me3 signal in aged ISC/EBs at *wor* (*C*) and enteroendocrine cells at *Ets21C* (*D*).

**Supplemental Figure S4.** Changes in H3K27me3 signal in regulatory regions in ISC/EBs and Enterocytes. *(A-B)* Log_2_ transformed counts and z-scored normalized heatmaps from differential analysis of H3K27me3 over STARR-seq enhancers (Zabidi et al. 2015) between young and old cells. *(A)* ISC/EBs have 3 enhancers that gain H3K27me3 signal with age and 15 that lose H3K27me3 signal with age. *(B)* Enterocytes have 264 enhancers that gain H3K27me3 signal with age and 142 that lose H3K27me3 signal with age. *(C-F)* Dot plots representing the -log_10_ q-value and percent of enhancers that contain a specific transcription factor motif from FIMO (Grant et al. 2011). *(C)* Motifs enriched in enhancers that lose H3K27me3 signal in aged ISC/EBs. *(D)* Motifs enriched in enhancers that gain H3K27me3 signal in aged ISC/EBs. *(E)* Motifs enriched in enhancers that lose H3K27me3 signal in aged enterocytes. *(F)* Motifs enriched in enhancers that gain H3K27me3 signal in aged enterocytes.

**
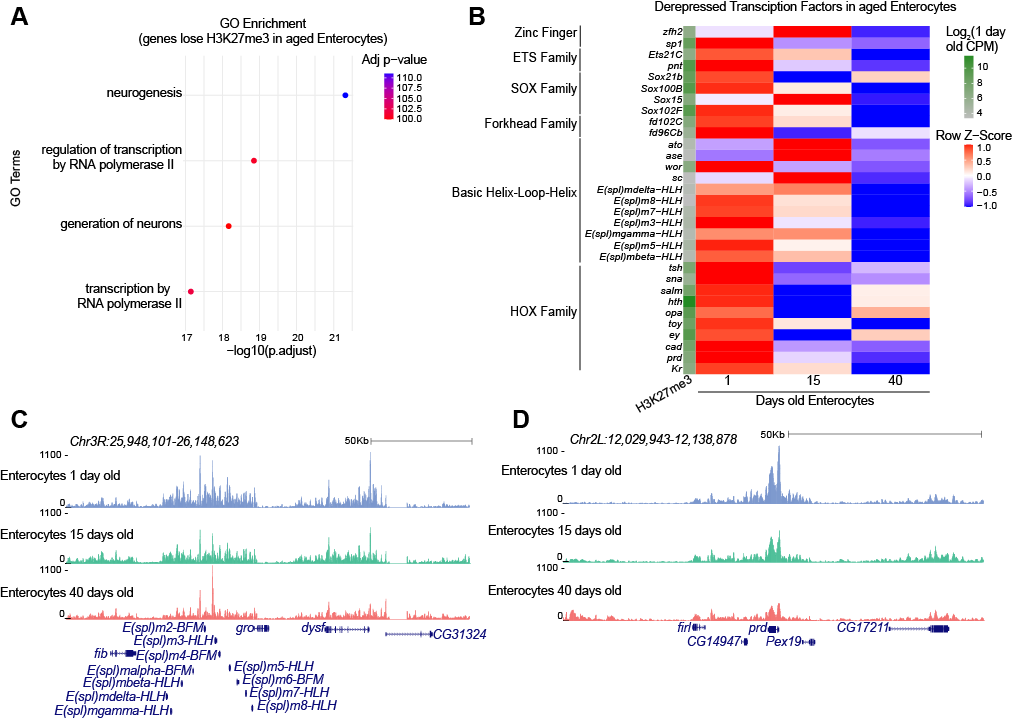
**

**Supplemental Figure S5.** Transcription factor genes lose the H3K27me3 mark in aged enterocytes. (*A*) Top four GO terms on genes that lose H3K27me3 signal in aged enterocytes from differential analysis. (*B*) Row *z*-score normalized heatmap of H3K27me3 counts for transcription factor genes identified in GO analysis from panel A that decrease in H3K27me3 signal in aged enterocytes. The first column is the log_2_ transformed counts for 1-day old tissue for each gene. (*C-D*) UCSC Genome Browser tracks of example transcription factors that lose H3K27me3 signal in aged enterocytes: *Enhancer of split* complex genes (*C*) and *paired* (*D*).

**
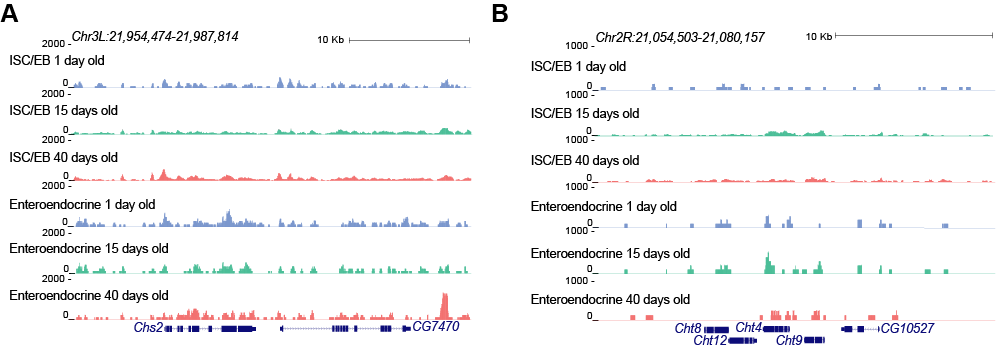
**

**Supplemental Figure S6.** Chitin genes lack the H3K27me3 mark in ISC/EBs and enteroendocrine cells. (*A-B*) UCSC Genome Browser tracks of H3K27me3 signal for ISC/EBs and enteroendocrine cells at *Chs2* (*A*) and cluster of *Cht* genes (*B*).

**Supplemental Figure S7.** Matrix of Pearson’s correlation coefficient for all RNAPIIS5P CUTAC reactions. Replicates between ages show high reproducibility.

**Supplemental Figure S8.** Genome browser tracks of RNAPIIS5P and Pc signal over chitin genes. *(A-B)* UCSC Genome Browser track of RNAPII5P over *Chs2 (A)* and cluster of *Cht* genes *(B)* for all three ages. *(C-D)* UCSC Genome Browser track of Pc over *Chs2 (C)* and cluster of *Cht* genes *(D)* for all three ages. *(E-F)* UCSC Genome Browser track of Pc over *rad* (*E*) and *dy* (*F*) for all three ages to illustrate low level of Pc signal that is not above background.


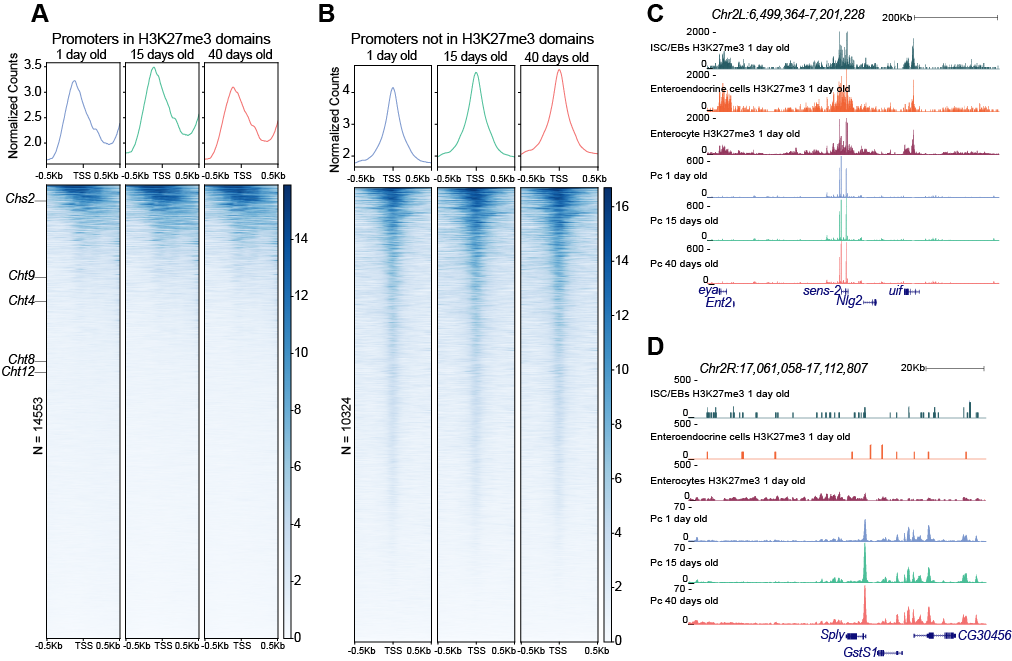


**Supplemental Figure S9.** Pc protein binds to promoters located inside and outside of H3K27me3 domains. (*A-B*) Summary plot and heatmap of Pc signal from three ages of guts aligned to transcription start site (TSS) of promoters located within

H3K27me3 domains (*A*) and promoters located outside of H3K27me3 domains (*B*). Pc is stably bound during aging. Chitin gene promoters are labelled in *A.* (*C-D*) UCSC Genome Browser tracks depicting Pc binding in the *sens-2* domain (*C*) and at the *Sply* promoter which lacks the H3K27me3 mark (*D*).

**Supplemental Figure S10.** Enteroendocrine precursor genes become derepressed in aged ISC/EBs. Row *z*-score normalized heatmap of H3K27me3 counts of ISC/EBs for precursor enteroendocrine and neural stem cells genes defined in (Tauc et al. 2021). The first column is the log_2_ transformed counts for 1-day old tissue for each gene. All of these genes become derepressed with age in ISC/EB.


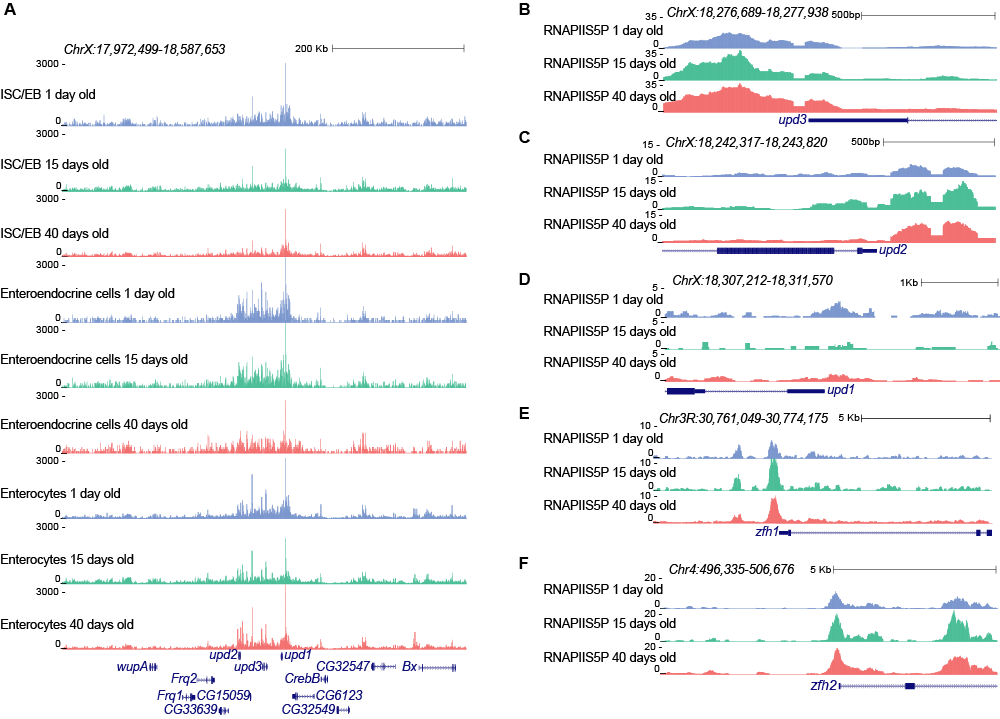


**Supplemental Figure S11.** Genome browser tracks of JAK/STAT ligands and target genes. *(A)* UCSC Genome Browser track of H3K27me3 level for all three cell types over the UPD domain. *(B-D)* UCSC Genome Browser tracks of RNAPIIS5P level over the promoters of *upd3 (B)*, *upd2 (C)*, and *upd1*. *(E-F)* UCSC Genome Browser tracks of RNAPIIS5P level over the promoters of known JAK/STAT targets genes such as *zfh1 (E)* and *zfh2* *(F)*.

**
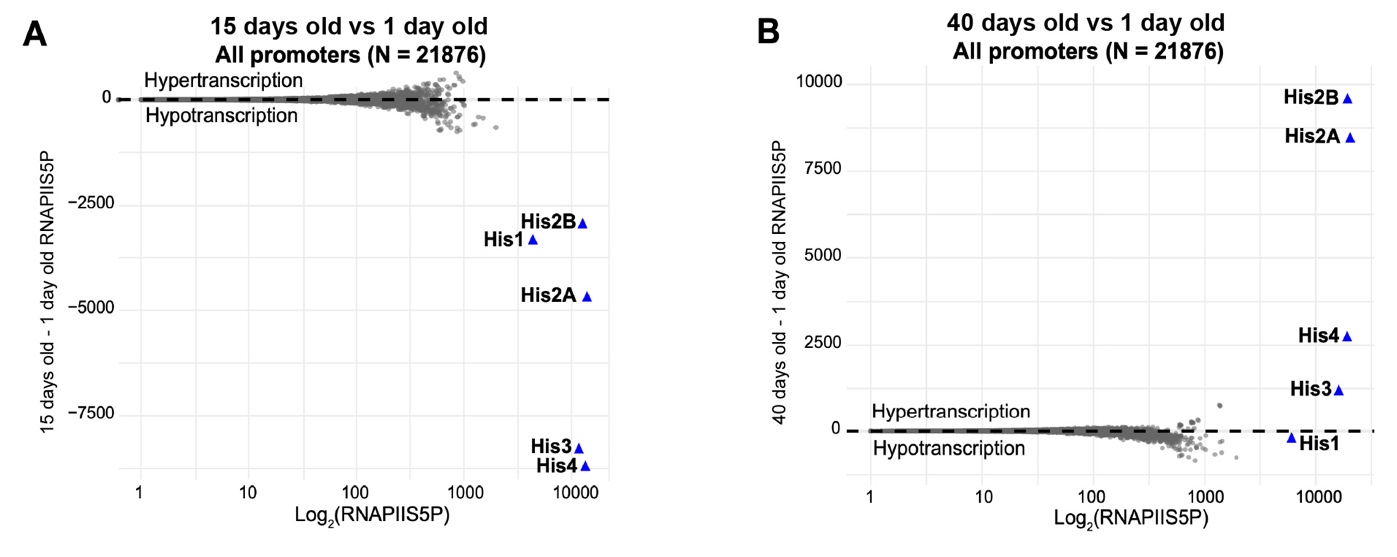
**

**Supplemental Figure S12.** S-phase-dependent histone genes gain RNAPIIS5P from 1 day old to 40 days old guts. (*A*) RNAPIIS5P counts for the absolute difference (15 days old – 1 day old) counts versus average count (log_10_(15 days old + 1 day old)/2) for all promoters in the Drosophila genome. The core histone genes are highly expressed and lose RNAPIIS5P in mid-age guts. (*B*) RNAPIIS5P counts for the absolute difference (40 days old – 1 day old) counts versus average count (log_10_(40 days old + 1 day old)/2) for all promoters in the Drosophila genome. The core histone genes gain RNAPIIS5P signal in 40 days old tissues**.**

**Supplementary Tables (provided as .xlsx):**

**Table S1:** Alignment statistics for sequencing data used in this paper

**Table S2:** List of peaks called by SEACR on aggregate H3K27me3 sciCUT&Tag data

**Table S3:** List of fragment numbers per cell for H3K27me3 sciCUT&Tag

**Table S4**: List of fraction of reads in peaks (FRiPs) per cell for H3K27me3 sciCUT&Tag

**Table S5**: List of ratio of reads that fall within blacklisted regions per cell for H3K27me3 sciCUT&Tag

**Table S6:** List of number of cells by cell type for each age and median fragments per cell

**Table S7:** List of genes that fall within H3K27me3 sciCUT&Tag peaks

**Table S8:** Gene count table for genes within peaks for ISC/EBs

**Table S9:** Gene count table for genes within peaks for enteroendocrine cells

**Table S10:** Gene count table for genes within peaks for enterocytes

**Table S11:** Results from differential analysis of genes between 1 day old cell types performed on the Degust server

**Table S12:** Results from differential analysis of genes between 1 day old and 40 days old cells for ISC/EBs performed on the Degust server

**Table S13:** Results from differential analysis of genes between 1 day old and 40 days old cells for enteroendocrine cells performed on the Degust server

**Table S14:** Results from differential analysis of genes between 1 day old and 40 days old cells for enterocytes performed on the Degust server

**Table S15:** Results from Gene Ontology analysis for genes that lose H3K27me3 signal in aged ISC/EBs

**Table S16**: Results from Gene Ontology analysis for genes that lose H3K27me3 signal in aged enteroendocrine cells

**Table S17:** Results from differential analysis for STARR-seq enhancers (Zabidi et al. 2015) between 1 day old and 40 days old ISC/EBs performed on the Degust server

**Table S18:** Results from differential analysis for STARR-seq enhancers (Zabidi et al. 2015) between 1 day old and 40 days old enterocytes performed on Degust server

**Table S19:** Results from Gene Ontology analysis for genes that lose H3K27me3 signal in aged enterocytes

**Table S20:** Results from Gene Ontology analysis for genes that gain H3K27me3 signal in aged enterocytes

**Table S21:** Cell type counts from single-cell RNA-seq data (Hung et al. 2020) for chitin genes

**Table S22:** Promoter count tables for RNAPIIS5P

**Table S23:** Cell counts from single-cell RNA-seq from (Tauc et al. 2021)

**Table S24:** Results from differential analysis of RNAPIIS5P at promoters between 1 day old and 40 days tissues performed on the Degust server

**Table S25:** Promoter count tables for Pc

**Table S26:** Pc signal vs IgG for selected promoters from Supplemental Figure S7

**References**

Grant CE, Bailey TL, Noble WS. 2011. FIMO: scanning for occurrences of a given motif. *Bioinformatics* **27**: 1017-1018.

Hung RJ, Hu Y, Kirchner R, Liu Y, Xu C, Comjean A, Tattikota SG, Li F, Song W, Ho Sui S et al. 2020. A cell atlas of the adult Drosophila midgut. *Proc Natl Acad Sci U S A* **117**: 1514-1523.

Tauc HM, Rodriguez-Fernandez IA, Hackney JA, Pawlak M, Ronnen Oron T, Korzelius J, Moussa HF, Chaudhuri S, Modrusan Z, Edgar BA et al. 2021. Age-related changes in polycomb gene regulation disrupt lineage fidelity in intestinal stem cells. *Elife* **10**: e62250.

Zabidi MA, Arnold CD, Schernhuber K, Pagani M, Rath M, Frank O, Stark A. 2015. Enhancer-core-promoter specificity separates developmental and housekeeping gene regulation. *Nature* **518**: 556-559.
